# Hepatocyte Growth Factor Reduces Free Cholesterol-Mediated Lipotoxicity in Primary Hepatocytes by Countering Oxidative Stress

**DOI:** 10.1155/2016/7960386

**Published:** 2016-04-10

**Authors:** Mayra Domínguez-Pérez, Natalia Nuño-Lámbarri, Denise Clavijo-Cornejo, Armando Luna-López, Verónica Souza, Leticia Bucio, Roxana U. Miranda, Linda Muñoz, Luis Enrique Gomez-Quiroz, Salvador Uribe-Carvajal, María Concepción Gutiérrez-Ruiz

**Affiliations:** ^1^Posgrado en Biología Experimental, DCBS, Universidad Autónoma Metropolitana Iztapalapa, 09340 México, DF, Mexico; ^2^Departamento de Ciencias de la Salud, Universidad Autónoma Metropolitana Iztapalapa, 09340 México, DF, Mexico; ^3^Unidad de Medicina Traslacional, Clínica y Fundación Médica Sur, 14050 México, DF, Mexico; ^4^Laboratorio de Sinovioanálisis, Instituto Nacional de Rehabilitación, 14389 México, DF, Mexico; ^5^Instituto Nacional de Geriatría, Secretaria de Salud, 10200 México, DF, Mexico; ^6^Red Fisiopatología de las Enfermedades Hepáticas, PRODEP-SEP, 09340 México, DF, Mexico; ^7^Unidad de Hígado “Dr. José E González”, Hospital Universitario, UANL, 66450 Monterrey, NL, Mexico; ^8^Departamento de Genética Molecular, Instituto de Fisiología Celular, Universidad Nacional Autónoma de México, 04510 México, DF, Mexico

## Abstract

Cholesterol overload in the liver has shown toxic effects by inducing the aggravation of nonalcoholic fatty liver disease to steatohepatitis and sensitizing to damage. Although the mechanism of damage is complex, it has been demonstrated that oxidative stress plays a prominent role in the process. In addition, we have proved that hepatocyte growth factor induces an antioxidant response in hepatic cells; in the present work we aimed to figure out the protective effect of this growth factor in hepatocytes overloaded with free cholesterol. Hepatocytes from mice fed with a high-cholesterol diet were treated or not with HGF, reactive oxygen species present in cholesterol overloaded hepatocytes significantly decreased, and this effect was particularly associated with the increase in glutathione and related enzymes, such as *γ*-gamma glutamyl cysteine synthetase, GSH peroxidase, and GSH-S-transferase. Our data clearly indicate that HGF displays an antioxidant response by inducing the glutathione-related protection system.

## 1. Introduction

Nowadays, lipid toxicity is gaining more attention due to the pandemic problem of obesity. Although every lipid possesses specific effects in cells and tissues, cholesterol is positioned as one of the more noxious lipids when it is overloaded in cells. An increase in the consumption of dietary cholesterol represents the main root to complications in the nonalcoholic fatty liver disease (NAFLD), such as nonalcoholic steatohepatitis (NASH) [1, 2]; in fact, interference in cholesterol synthesis has proved to induce protective effects against ischemia-reperfusion injury; in addition, cholesterol overload in hepatocytes sensitizes to damage mediated by death receptors ligands such as TNF-*α* and FAS [2].

The mechanism of cholesterol-induced cell toxicity is not well understood. Some evidences indicate disturbances in cell plasma membrane [3] and others changes in redox homeostasis by inducing the activation of prooxidant systems such as NADPH oxidase, which, in addition, increases reactive oxygen species (ROS) production, as we recently reported [4], or depletion in mitochondrial glutathione (GSH) [2].

It has been widely reported that the hepatocyte growth factor (HGF) displays a response that regulates the redox homeostasis by different mechanisms and diverse liver diseases such as the expression of antioxidant proteins, or repression of prooxidant systems in alcoholic liver disease [5–8], in drug-induced liver injury [9, 10], or by the action of cytotoxic growth factors like transforming growth factor beta (TGF-*β*) [11]; even more, HGF can suppress the activity and expression of one of the main ROS generator systems, the NADPH oxidase [12].

The antioxidant response of HGF and the other canonical functions such as proliferation, antiapoptosis, or motogenesis are triggered by its receptor c-Met [13–15]. More evidence have shown that c-Met signaling abrogation, by genetic interference in the receptor, leads to oxidative stress-associated disease progression or aggravation, particularly in fibrosis [16], apoptosis mediated by death receptors [17], or chemical-induced hepatocarcinogenesis [18]; in all cases c-Met KO mice exhibited oxidative stress, even, in the absence of any other stimulus.

In the present work, we were focused on figuring out the antioxidant effect of the HGF in the cholesterol-induced toxicity in primary mouse hepatocytes, using a dietary model of cholesterol liver overload.

## 2. Material and Methods

### 2.1. Materials

2′,7′-Dichlorodihydrofluorescein diacetate (DCFH) was purchased from Invitrogen (catalog number c-6827) and recombinant human HGF from PeproTech (Rocky Hill, NJ). All other chemicals were purchased from Sigma-Aldrich (San Louis, MO).

### 2.2. Mice, Hepatocyte Isolation, and Culture

CD1 male mice (8 to 10 weeks old) were maintained in pathogen-free housing and cared for in accordance with the Universidad Autónoma Metropolitana and NIH Guide for the Care and Use of Laboratory Animals.

Twenty mice were randomly divided in two groups; in HC group 10 animals were fed with a high-cholesterol diet (HC, 2% cholesterol and 0.5% sodium cholate) for two days, as reported by Marí and coworkers [2]. Ten control animals were fed with a regular rodent Chow diet (Purina #5001). After the two days under HC and Chow diets, five animals were sacrificed.

Hepatocytes were isolated from the rest of the HC and Chow mice by the two-step collagenase perfusion, as we previously described [12]. The viability was >90% as assessed by trypan blue exclusion. Hepatocytes were seeded at 2.13 × 10^5^ cells per cm^2^ either in Lab-Tek chambered slides or in 10 cm dishes (Nalge, Nunc) in the Ham's F-12/Dulbecco's modified Eagle's basal hepatocyte growth medium supplemented with 10% fetal bovine serum. After 4 h of cells attachment, media were replaced by a serum-free basal hepatocyte growth medium. In the following day, cells were treated with 50 ng/mL HGF.

### 2.3. Biochemical Determinations, HGF Serum Content, and Analysis of Liver Function

Serum levels of cholesterol, aspartate aminotransferase (AST), alanine transferase (ALT), and alkaline phosphatase (ALP) activities were determined by automated method using Reflovet Plus (Roche).

Serum samples were obtained from NASH patients and healthy volunteer donors (referred to as control), in the Liver Unit at the University Hospital (UANL, Monterrey, NL, Mexico). All participants signed the document of informed consent process. Serum content of HGF was addressed by ELISA assay using either Mouse/Rat or human HGF Quantikine Immunoassays (R&D systems, Minneapolis, MN), following manufacturer's instructions.

### 2.4. Western Blot Analysis

Western blot analysis was performed as we previously described [17] using antibodies listed in Supplementary Table 1, in Supplementary Material available online at http://dx.doi.org/10.1155/2016/7960386.

### 2.5. Protein Content Determination

Total protein concentration was determined using the bicinchoninic acid (BCA) kit (Pierce Thermo Scientific, Rockford, IL), according to the manufacturer's instructions.

### 2.6. Oxidative Stress Parameters

Reactive oxygen species (ROS) content was determined using 2′-7′-dichlorodihydrofluorescein diacetate (DCFH), as previously reported [12]. Hepatocytes from both animals Chow and HC were seeded in 96-well plates (5 × 10^4^ cells/well); after overnight stabilization cells were incubated with 5 *μ*M DCFH, a cell-permeable nonfluorescent probe that is deesterified intracellularly and converted to the highly fluorescent 2′,7′-dichlorofluorescein upon oxidation by ROS, particularly peroxides (H_2_O_2_); fluorescence was detected using a DTX 880 multimodal detector (Beckman Coulter) with excitation wavelength of 480 nm and emission wavelength of 520.

Carbonyl modification of proteins is a key biomarker for the identification of oxidative stress and was addressed by using Oxyblot Protein Oxidation Detection Kit (Millipore, Darmstadt, Germany).

SOD and catalase enzymes activities were determined as we previously reported [19], and GSH and GSSG content were assayed by HPLC as previously reported [17].

### 2.7. Histology and Immunohistochemistry

Formalin-fixed paraffin-embedded liver sections were stained with hematoxylin and eosin (H&E) following standard procedures.

### 2.8. c-Met Immunofluorescence

Immunofluorescence of c-Met was determined as previously reported [20] using anti-c-Met antibody (Cell Signaling Inc.).

### 2.9. Free Cholesterol Determination by Filipin Staining

Isolated hepatocytes were fixed with formalin 10% for 1 h at room temperature. For free cholesterol determination, cells were incubated with filipin 0.2 mg/mL overnight at 4°C protected from light. After 3 final washes in PBS, cells were mounted and confocal microscopy images were collected using UV light excitation.

### 2.10. Neutral Lipid Determination by Oil Red O (ORO)

Neutral lipids were determined by ORO staining as previously reported [21]. Fixed hepatocytes were stained with 0.2% ORO solution for 4 h. After rinsing with PBS, cells were counterstained with hematoxylin.

### 2.11. Statistical Analysis

The data are presented as mean ± SEM for at least three independent experiments carry out by triplicate. Comparisons between groups were made using Student's *t*-test, and Mann-Whitney test. GraphPad Prism 6 software for OSX was used to run analysis. Differences were considered significant at *p* ≤ 0.05.

## 3. Results

### 3.1. A HC Diet Induces Steatosis and Liver Damage

**Figure 1:**
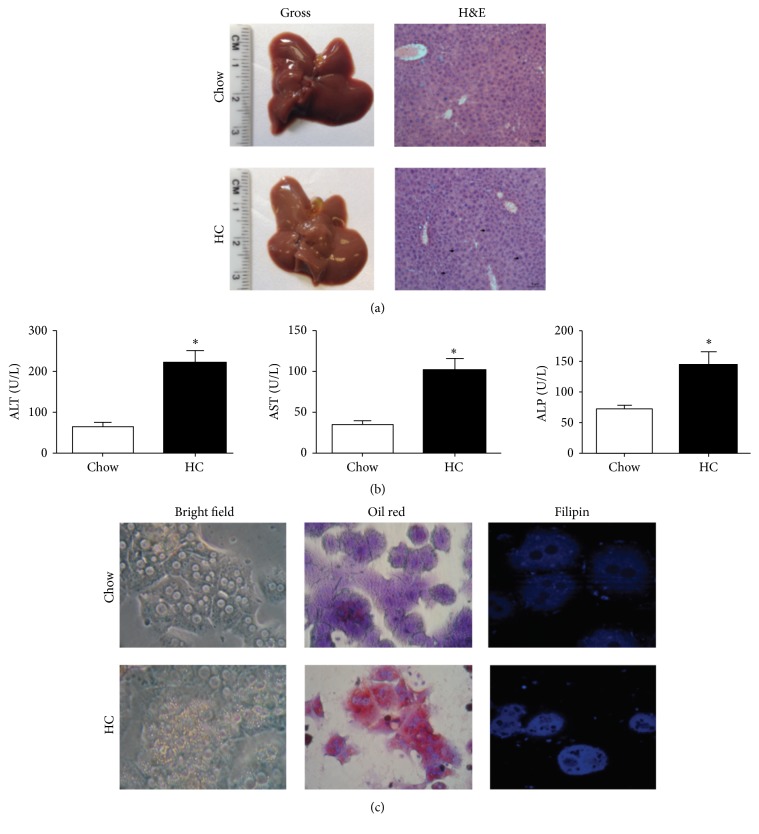
High-cholesterol diet induces liver injury and hepatocytes lipid overload. (a) Gross inspection of the liver and hematoxylin and eosin (H&E) staining of liver tissue from animals under normal Chow and high-cholesterol (HC) diet for two days. Arrows indicate hepatocyte proliferation. (b) Liver function test: alanine aminotransferase (ALT), aspartate aminotransferase (AST), and alkaline phosphatase (ALP). (c) Bright filed microscopy; neutral lipids and free cholesterol content assessed by Oil Red and filipin staining, respectively, of HC and Chow hepatocytes. Images are representative of at least three independent experiments. Original magnification, 200x. Each column represents mean ± SEM of at least four independent experiments. ^*∗*^ *p* < 0.05 versus Chow diet.

Animals were fed with a high-cholesterol diet for two days; Figure 1(a) shows gross inspection of the livers; HC exhibits the characteristic pale color of steatosis, and a gallbladder increased in size, in comparison with animals fed with Chow regular diet. Routine H&E staining revealed a microvesicular steatosis and high number of binucleated hepatocytes (arrows) in HC tissue, suggesting ongoing repair process after tissue damage.

The analysis of liver damage markers reveals hepatocellular injury due to HC diet increasing the ALT, AST, and ALP activity in serum from animals under that diet (Figure 1(b)).

Hepatocytes isolated from HC mice and cultured for 24 h exhibited an increment in lipid content, as bright field microscopy clearly shows (Figure 1(c)). Neutral lipids and free cholesterol determination by ORO and filipin staining, respectively, confirmed lipid content in comparison with cells from animals fed with Chow diet; the lipid overload was observed even with bright field microcopy; these results indicate that hepatocytes are overloading lipids and cell culture did not affect this feature.

### 3.2. Lipid Accumulation in the Liver Decreases c-Met Content in Plasma Membrane and HGF Concentration in Serum

**Figure 2:**
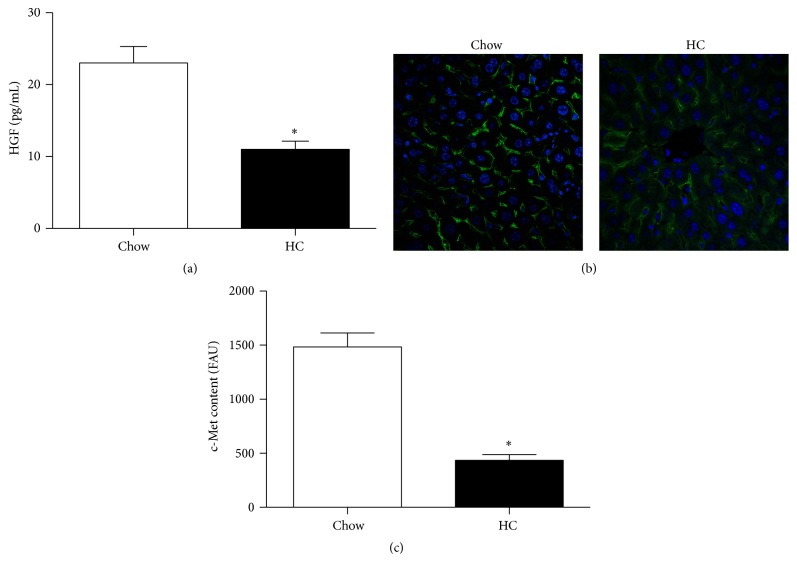
*Cholesterol overload in primary mouse hepatocytes affects HGF levels and c-Met expression*. A high cholesterol diet induces a decrease in serum HGF levels (a), quantified by ELISA, and c-Met expression assayed by immunofluorescence, original magnification, 200X (b). c-Met immunofluorescence quantification (c). Images are representative of at least three independent experiments. Each column represents mean ± SEM of at least four independent experiments. ^*∗*^ *p* < 0.05 versus Chow.

Due to HGF and c-Met transducing the main repair and survival signals in the damaged liver [13], we decided to address the HGF serum levels in animals under both diets.  Figure 2(a) shows a significant decrease in serum HGF concentration in HC animals, and c-Met content in plasma membrane is considerably decreased in HC liver tissue as judged by immunofluorescence of the receptor (Figure 2(b)).

### 3.3. Hepatocytes from HC Fed Animals Are under Oxidative Stress

One of the main consequences of cell lipid overload is ROS generation. Supplementary Figure 1 shows that HC hepatocytes are overproducing ROS judged by DCFH fluorescence (Supplementary Figures 1A and B), and it was related to an increment in protein oxidation (Supplementary Figure 1C) when comparing with Chow cells.

### 3.4. HGF Decreases ROS Content in HC Hepatocytes by Increasing GSH Antioxidant System

**Figure 3:**
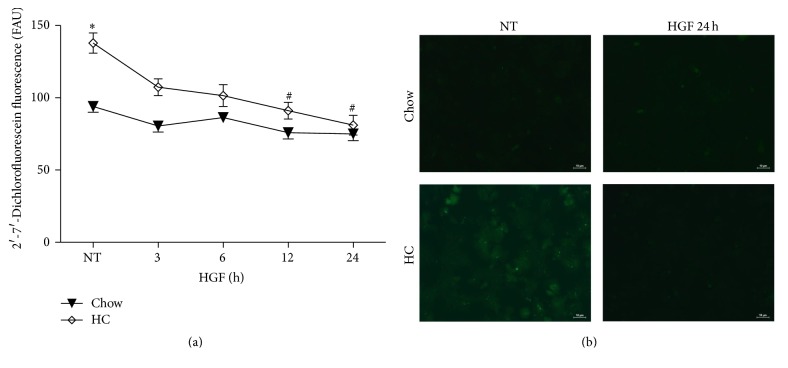
*HGF decreases the oxidative stress induced by the cholesterol overload.* (a) Quantification of 2′,7′-dichlorofluorescein fluorescence: data are reported as fluorescence arbitrary units (FAU). Each point represents mean ± SEM of at least four independent experiments. (b) Representative confocal images of peroxides content determined by 2′,7′-dichlorofluorescein fluorescence in Chow and HC cells treated or not with HGF for 24 h. ^*∗*^ *p* < 0.05 versus Chow, ^#^ *p* < 0.05 versus nontreated HC cells.

**Figure 4:**
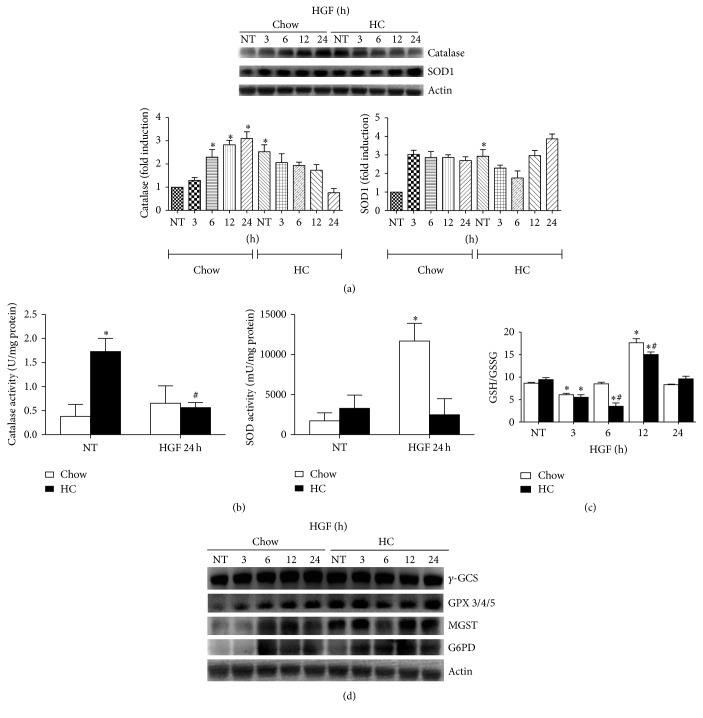
HGF effect on antioxidants enzymes and GSH ratio. (a) Western blot and densitometric analysis of catalase and superoxide dismutase 1 (SOD1) under HGF treatment for different times. (b) Enzymatic activity of catalase and SOD, in Chow and HC cells treated with HGF for 24 h. (c) Ratio of reduced glutathione (GSH) to oxidized glutathione (GSSG), determined by HPLC. Chow and HC cells were treated with HGF for different times. Each column represents the mean ± SEM of three independent experiments. (d) Western blot analysis of *γ*-gamma glutamyl cysteine synthetase (*γ*-GCS); GSH peroxidase (GPX) 3/4/5; mammal GSH-S-transferase (MGST); and glucose-6-phosphate dehydrogenase (G6PD). Images are representative of at least three independent experiments. Actin was used as housekeeping loading control. Each column represents mean ± SEM of at least four independent experiments. ^*∗*^ *p* < 0.05 versus nontreated Chow cells; ^#^ *p* < 0.05 versus nontreated HC cells.

As previously reported HGF can control cellular redox status [12]; to figure out if HGF is able to induce this response in cholesterol overloaded cells, we treated hepatocytes with HGF (50 ng/mL) for different times and ROS content was determined.  Figure 3(a) shows that HGF decreased ROS in HC hepatocytes in a time-dependent manner, reaching control values at 24 h; this effect was corroborated by fluorescence microscopy (Figure 3(b)) at 24 h. HGF induced the expression of catalase at 12 and 24 h in Chow cells. SOD1 was also increased in HC cells but failed in Chow hepatocytes (Figure 4(a)). To confirm that, we assayed the activity of these enzymes at 24 h of HGF treatment; Figure 4(b) shows the activity of the enzymes; catalase activity is considerably increased in HC NT cells, in comparison with Chow NT, but no changes were observed in HGF treatment; in the case of total SOD activity there is no difference in NT cells, and HGF only induced an increase in the activity in Chow cells.

To gain more evidence of the HGF-induced antioxidant response, we assayed the GSH ratio by HPLC; Figure 4(c) depicts an increase in GSH ratio at 12 h of treatment, which is consistent with the cellular response elicited by HGF. The immunoblot analysis of the main GSH-related antioxidant enzymes (Figure 4(d)) shows that HGF was able to induce the expression of *γ*-GCS, G6PD, MGST, and GPX3/4/5 in Chow cells; interestingly no significant changes were observed in *γ*-GCS in HC hepatocytes; however, G6PD was elevated in HC suggesting GSSG recycling as a mechanism of protection (Supplementary Figure 2).

**Figure 5:**
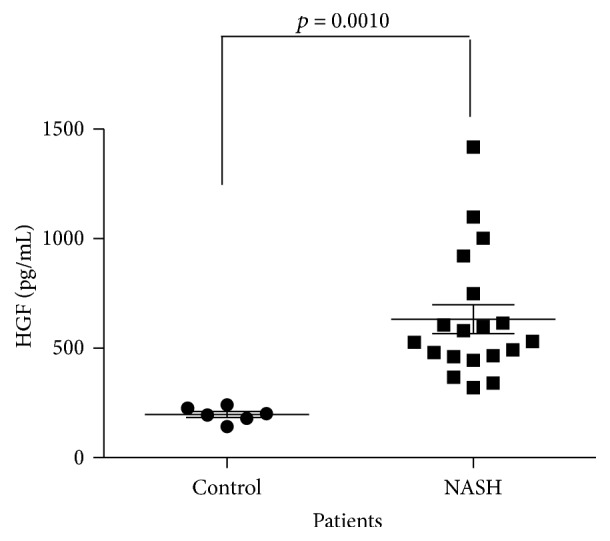
Serum HGF levels in NASH patients and healthy volunteers (control) determined by ELISA.

Finally, in order to find the significance of HGF in cholesterol-relevant human liver disease, such as nonalcoholic steatohepatitis in humans [1], we assayed serum HGF levels in patients with NASH.  Figure 5 shows the ELISA result of HGF quantification; it is clear that NASH patients present a significant increase in circulating levels of HGF; these data suggest that HGF/c-Met signaling is required for a proper response against NASH in humans where free cholesterol is frequently accumulated.

## 4. Discussion

We, and others, have previously reported that cholesterol overload induces cytotoxic effects and oxidative stress in the liver [2, 4, 22], as Figure 1 supports; although Marí and collaborators reported that mitochondrial GSH depletion is related to sensitization to TNF- or FAS-induced cellular damage [2], the mechanism of oxidative stress-mediated injury remained partially characterized.

It has been extensively reported that HGF displays an antioxidant response under canonical liver insults, inducing protection by the expression of antioxidant [8], and antiapoptotic proteins [17]. In order to characterize the response of HGF in cholesterol overloaded hepatic cells, we isolated primary mouse hepatocytes from animals fed with a high-cholesterol diet for two days. Although the dietary model that we followed is not canonical of NASH, primary mouse hepatocytes from HC animals clearly exhibited an increment in neutral lipids and free cholesterol content, as Figure 1(c) shows; in addition, oxidative stress was also confirmed (Supplementary Figure 1); our results in mice are in agreement with those found in rats by Marí and coworkers [2].

HGF efficiently displays an antioxidant response by increasing key enzymes that maintain ROS under control [23], such as catalase or SOD1, in a mechanism dependent on the activation of nuclear factor kappa B [5]. It is important to note that alcohol-induced oxidative stress is dependent on the activity of the cytochrome P450 2E1 located in the endoplasmic reticulum; in the steatosis model used in this study, mitochondria play a significant role [2], and we were focused to know if HGF can also display a protective response in cells with mitochondria dysfunction as it does control NADPH oxidase- or Cyp2E1-mediated damage, in hepatic cells [5].  Figure 3(a) shows that HGF treatment decreases ROS production and protein oxidation. Interestingly, catalase content, determined by western blotting, was induced in hepatocytes from Chow fed animals, but its content decreased in a time-dependent manner in HC cells; even more, SOD1 increases in HC cells, with no significant changes in Chow hepatocytes (Figure 4(a)).

To confirm our data, we decided to assay the activity of both enzymes at 24 h of HGF treatment. Cholesterol induced 4-fold increment catalase activity and HGF treatment decrease it to basal values; this result is in agreement with that observed in western blot. In the case of SOD, no changes were observed in the activity of the enzyme under cholesterol treatment; however, as expected, HGF induced the SOD activity at 24 h. These proteins are among the main ROS detoxifying enzymes, and particularly the three forms of SOD are quite sensitive to ROS changes inducing their content decrement or activity as we recently showed [24]; however, cholesterol overload in hepatocytes does not seem to alter SOD at least at two days under HC diet.

To continue exploring the antioxidant response elicited by HGF, we were focused on the GSH system. We have clear evidence that HGF can stimulate the GSH system machinery, particularly the key enzyme in GSH synthesis, the gamma-glutamylcysteine synthetase (*γ*-GCS), which, in addition, increases GSH content [5, 17]; in the present study we did find the same effect in Chow cells; interestingly, *γ*-GCS content in HC cells slightly decreased with HGF treatment, but GSH/GSSG ratio presented a different behavior; HGF induced a decrease in the tripeptide ratio at 6 h comparing with Chow cells, and at 12 h GSH ratio exhibited a significant recovery. These were in agreement with the expression of the main enzymes related to GSH, such as *γ*-GCS, GSH peroxidase, GSH-S-transferase, and glucose-6-phosphate dehydrogenase, which provide NADPH, the main electron donor in the reduction of GSSG into GSH by GSH reductase [25]; all these enzymes were induced by HGF in the Chow cells, in a time-dependent manner; nontreated HC cells exhibited a basal overexpression of these antioxidant enzymes; comparing with nontreated Chow cells, a slight increment was observed at 12 and 24 h. These data clearly indicate that HGF protects by inducing GSH systems. As indicated by Marí and coworkers in rat hepatocytes [2], mitochondrial GSH depletion induced the sensitization to death receptors-mediated injury; the reestablishment of GSH avoided this effect. Similarly it has been observed that c-Met signaling deletion triggered more liver lesions in animals under N-nitrosodiethylamine treatment [18]; the coadministration of N-acetyl-cysteine, a well known GSH precursor [26], induced a significant decrease in lesion formation and in size of tumors, leaving the relevance of GSH in HGF-mediated cellular protection clear.

The relevance of HGF in lipid-induced toxicity has been confirmed even in nonhepatic cells such as the insulinoma-derived cell line RINm5F, where the growth factor repressed the free fatty acid-induced apoptosis by counteracting oxidative stress [15]. In addition, the specific c-Met elimination in the liver presented an aggravated steatosis under methionine-choline deficient diet, compared with WT litter mice [27], characterized for an enhanced apoptosis and inflammatory response.

Finally, it is clear that HGF induces an antioxidant response; however, it is well known that this growth factor can control many other cellular responses. As it has been proved in the present work, high-cholesterol diet can lead to oxidative stress, characteristic that can aggravate the liver disease, such as steatosis or steatohepatitis. More research is required to understand the impact of HGF, even far away from ROS manage.

## Supplementary Material

For Western blot analysis we used the following primary antibodies listed in supplementary figure 1, followed of proper secondary antibodies conjugated with horseradish peroxidase.
The HC diet induces oxidative stress, judged by peroxides content identified by DCFH fluorescence (supplementary figure 1 a and b) and by protein oxidation addressed by oxyblot (supplementary figure 1 c).
From figure 3 panel (d) we performed a densitometric analysis of the Western blots. The result of this analysis is depicted in supplementary figure 2.
