# Supplementary material for: Hepatocyte Growth Factor Reduces Free Cholesterol-Mediated Lipotoxicity in Primary Hepatocytes by Countering Oxidative Stress
Source: Oxid Med Cell Longev. 2016 Apr 10;2016:7960386. doi: 10.1155/2016/7960386 (PMC4842075; doi:10.1155/2016/7960386)
Supplement: Supplementary file 1 — For Western blot analysis we used the following primary antibodies listed in supplementary figure 1, followed of proper secondary antibodies conjugated with horseradish peroxidase. The HC diet induces oxidative stress, judged by peroxides content identified by DCFH fluorescence (supplementary figure 1 a and b) and by protein oxidation addressed by oxyblot (supplementary figure 1 c). From figure 3 panel (d) we performed a densitometric analysis of the Western blots. The result of this analysis is depicted in supplementary figure 2. [file 7960386.f1.pdf]

Supplementary information and data

Antibody	Catalog	Company	Dilution
Catalase	C0979	Sigma Aldrich	1:3000
SOD 1	sc-11407	Santa Cruz Biotechnology	1:2000
γ-GCS	sc-22755	Santa Cruz Biotechnology	1:1000
MGST	sc-138	Santa Cruz Biotechnology	1:200
GPX 3/5/6	sc-55102	Santa Cruz Biotechnology	1:200
G6PD	8866	Cell Signaling	1:1000
Actin	A3854	Sigma Aldrich	1:10,000

Supplementary table 1. Antibodies used in Western blots studies in the present work SOD, Superoxide dismutase; g-GCS, g-glutamylcysteine synthetase; MGST, mammal Glutathione S transferase; GPX, Glutathione peroxidase. G6PD, Glucose-6-Phosphate Dehydrogenase

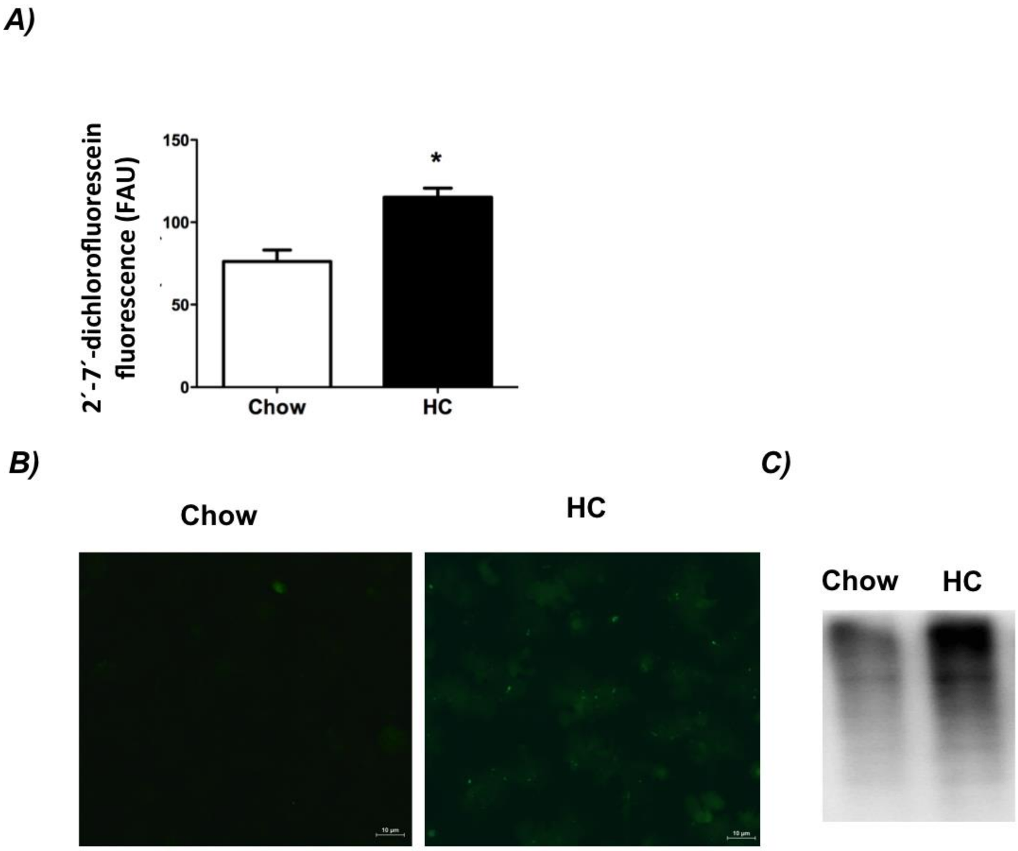

Supplementary figure 1 *Cholesterol induces oxidative stress in primary mouse hepatocytes*. Peroxides content determined by DCFH fluorescence in, (A) chow and, (B) HC hepatocytes. (C) Quantification of 2′,7′-dichlorofluorescein fluorescence, data are reported as fluorescence arbitrary units (FAU). (D) Protein oxidation determined by oxyblot. Images are representative of at least three independent experiments. Each column represents mean ± SEM of at least four independent experiments. * $p < 0.05$ vs chow diet.

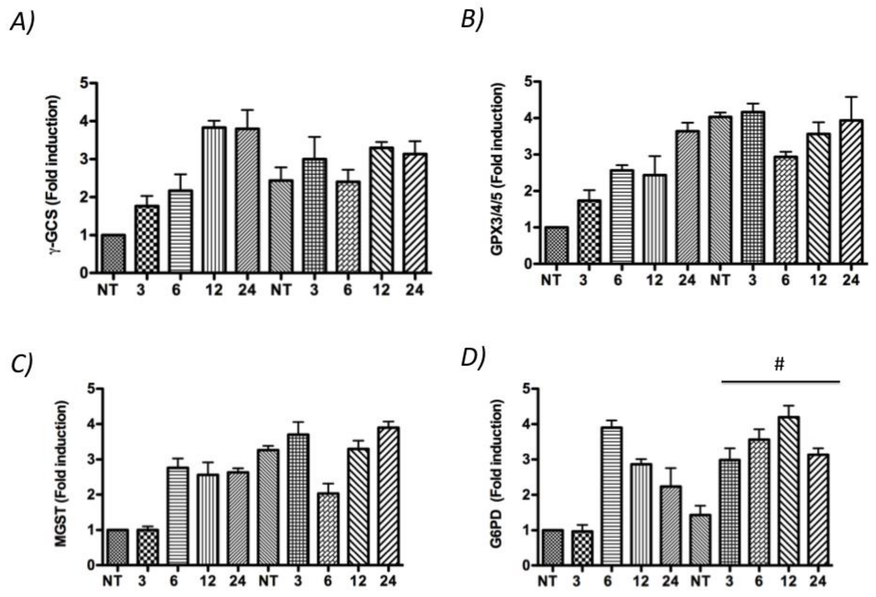

Supplementary figure 2 *HGF effect on antioxidants enzymes.* Densitometric analysis of Western blots of figure 4. (A) γ- gamma glutamyl cysteine sinthetase (γ-GCS); (B) GSH peroxidase (GPX) 3/4/5; (C) mammal GSH S transferase (MGST); and (D) glucose-6-phosphate dehydrogenase (G6PD). Each column represents mean ± SEM of at least four independent experiments. * $p < 0.05$ vs not treated Chow cells, # $p < 0.05$ vs not treated HC cells.
